# Supplementary material for: A switch in surface polymer biogenesis triggers growth-phase-dependent and antibiotic-induced bacteriolysis
Source: eLife. 2019 Apr 9;8:e44912. doi: 10.7554/eLife.44912 (PMC6456293; doi:10.7554/eLife.44912)
Supplement: Supplementary file 1. [file elife-44912-supp1.docx]

**Supplementary File 1. Table of oligonucleotides used in this study**

| **Name** | **Sequence** |
| --- | --- |
| AB_Marker_F | GAGGGAGGAAAGGCAGGA |
| AB_Marker_R | CGCCGTATCTGTGCTCTC |
| BgaA_5FLANK_F | CCTACATTTGATGACCTTCTTAACGCC |
| BgaA_5FLANK_R | AACTTCGTCAGTGTCGCCTTGC |
| *bgaA*_FLANK_F | GTTGCTACTAAACGTCTTCAAGGACG |
| *bgaA*_FLANK_R | CGCCAAACTTCAAGAACAGATACAGG |
| pLEM023_F | CACGATTACTTGGGGATCTCCCCGCGAAAGCGGG |
| pLEM023_R | TCCTGCCTTTCCTCCCTCGTCATACCATGTATACCACTTGG |
| *lytA*_5F_F | GATGAGTTCAATTGTATCTATCGGCAGTG |
| *lytA_*5F_R | TCCTGCCTTTCCTCCCTCCTACTCCTTATCAATTAAAACAACTCATTTTTTACAATCC |
| *lytA*_3F_F | GAGAGCACAGATACGGCGCCAGATGGCTTGATTACAGTAAAATAATAATGG |
| *lytA*_3F_R | CTCAATCTATATAACATAGCTTTATGACTGATACC |
| *lytA*_5F_SEQ | GGACTTGCTACCATTATTTCGCAAGG |
| *tacL*_5F_F | GACTTGGCTTCAATTAGAAAAAGG |
| *tacL* _5F_R | TCCTGCCTTTCCTCCCTCGCCTATTGATTTCAAAATGAATCC |
| *tacL* _3F_F | G GAGAGCACAGATACGGCGGGCTTTATTTTCTTGCTAGTTATGTGC |
| *tacL* _3F_R | TGCGTGTTTTAAACCGTTCACGAAATCTTTAGC |
| *tacL* _5F_SEQ | GGACTCTGCCTTTGTTCCAATACAACTACGCAGGC |
| *ftsH*_5F_F | CAACAAATACCTGTTTCACGTGAAACATCC |
| *tacL* _5F_R | TCCTGCCTTTCCTCCCTCGAATACACTTCCTCTTTCAATACTCCAATATAAGG |
| *ftsH* _3F_F | GAGAGCACAGATACGGCGGAAAAATAACCCTGAGAGAGGCTGG |
| *ftsH* _3F_R | CAGTACTACAACTTATGTTGTAGCGCC |
| *ftsH* _5F_SEQ | TCCGCAGGCTGATGAAAAGG |
| *tacL*_F_nativeRBS_XhoI | TATCTCGAGGGTACAATATAGGATAGCTTACTATTATCTGAATCAGC |
| *tacL*_R_BamHI | ATAGGATCCCAAACTTATAAAACTTAATCCGTCATGTCCGATACC |
| *tacL*_FLAG | TTACTTGTCGTCATCGTCTTTGTAGTCATCCGTCATGTCCGATACCAACATTCGATGC |
| AB_marker_FLAG_F | GACTACAAAGACGATGACGACAAGTAAGAGGGAGGAAAGGCAGGA |
| *tacL*_ABmarker_F | GAGAGCACAGATACGGCGGTTTTATAAGTTTGAAATCTTCTACC |
| *lytA* _H26A_F | AGGCAAGTACACGCAGCCTCAACTGGGAATCCG |
| *lytA* _H26A_R | CGGATTCCCAGTTGAGGCTGCGTGTACTTGCCT |
| *lytA*_ABmarkerF_R | TCCTGCCTTTCCTCCCTCCCATTATTATTTTACTGTAATCAAGCCATCTGG |
| *lytA*_3F_F_ABmarker_R | GAGAGCACAGATACGGCGAATGTCTTTCAAATCAGAACAGCGCATATTATTAGGTC |
| *lytA*_F_purification_NdeI | GCCCATATGGAAATTAATGTGAGTAAATTAAGAACAGATTTGCC |
| *lytA*_R_purification_HindIII | GCCAAGCTTTTATTTTACTGTAATCAAGCCATCTGGCTCTACTGTGAATTCTGG |
